# Association of Perceived Lack of Paternal Support After Stillbirth With Maternal Postpartum Depression or Anxiety

**DOI:** 10.1001/jamanetworkopen.2022.31111

**Published:** 2022-09-12

**Authors:** Adam K. Lewkowitz, Tess E. K. Cersonsky, Uma M. Reddy, Robert L. Goldenberg, Donald J. Dudley, Robert M. Silver, Nina K. Ayala

**Affiliations:** 1 Department of Obstetrics and Gynecology, Alpert Medical School of Brown University, Providence, Rhode Island; 2 Department of Medical Education, Alpert Medical School of Brown University, Providence, Rhode Island; 3 Department of Obstetrics & Gynecology, Columbia University School of Medicine, New York, New York; 4 Department of Obstetrics & Gynecology, University of Virginia School of Medicine, Charlottesville; 5 Department of Obstetrics and Gynecology, University of Utah School of Medicine, Salt Lake City

## Abstract

This cross-sectional study examines the incidence of postpartum depression and anxiety in women who perceive a lack paternal support after stillbirth.

## Introduction

Stillbirth is associated with increased risk of postpartum depression or anxiety (PPDA).^1^ A father’s refusal to discuss stillbirth has been associated with increased maternal risk for PPDA,^2^ and perceived social support reduces PPDA risk after live birth.^3^ We examined the association between perceived paternal support following stillbirth and maternal PPDA.

## Methods

The Stillbirth Collaborative Research Network conducted a case-control study in 59 hospitals in 5 US catchment areas from 2006 to 2009. Data analysis for the present study was performed in May 2022. The study was approved by each collaborating site’s institutional review board. Study design, methods, and recruitment have been reported.^4^ Sociodemographic, medical, and psychosocial variables were collected. Women from the parent study who had a stillbirth, consented for future studies, and were not lost to follow-up were contacted 6 to 36 months after delivery to participate in a telephone interview containing validated psychometric instruments assessing PPDA.^4^ We followed the STROBE reporting guideline for case-control studies. This study population was limited to participants who responded to interview questions pertaining to paternal support, defined by the study participant responding to yes or no questions about perceiving support from the stillborn infant’s father.

The main outcome was PPDA, defined as a score of 13 or higher on the Edinburgh Postnatal Depression Scale^5^ and/or a score of 40 or higher on the State-Trait Anxiety Inventory for Adults.^6^ Outcomes were compared between women who did and did not perceive paternal support. Multivariate logistic regression controlled for PPDA risk factors (self-reported race and ethnicity, history of depression or anxiety, and substance use in pregnancy). Adjusted odds ratios (aORs) with 95% CIs were calculated to compare the absence of perceived paternal support with the odds of risk factors for maternal PPDA after stillbirth. Analyses were completed in R, version 4.1.3 (R Foundation for Statistical Support). With 2-sided, unpaired analysis using the Wilcoxon rank sum test, the significance threshold was *P* < .05.

## Results

Of 663 women with stillbirth in the parent study, 275 (41.5%) participated in the follow-up interview. Of 269 women who answered paternal support questions, 238 (88.5%) indicated they received support. Women who did vs did not perceive paternal support after stillbirth were similar in terms of median age (28.4 vs 27.3 years; *P* = .31), non-White race (76 [31.9%] vs 8 [25.8%]; *P* = .63), Hispanic ethnicity (68 [28.6%] vs 14 [45.2%]; *P* = .09), and alcohol (83 [34.9%] vs 15 [48.4%]; *P* = .25) or tobacco (27 [11.3%] vs 6 [19.4%]; *P* = .25) use during pregnancy.

Rates of PPDA were high overall (49.8%). However, women who did not perceive paternal support following stillbirth had more than 4 times higher odds of experiencing PPDA vs those who received support (25 [80.6%] vs 109 [45.8%]; aOR, 4.67; 95% CI, 2.73-8.00) (Table 1). Similarly, aORs of postpartum depression (aOR, 2.23; 95% CI, 1.46-3.41) and anxiety (aOR, 2.73; 95% CI, 1.75-4.26) alone were higher among women who did not perceive paternal support. In independent analysis, an absence of perceived support from the stillborn infant’s father was associated with higher odds of maternal PPDA (aOR, 4.32; 95% CI, 2.46-7.60) than other PPDA risk factors, including prepregnancy history of depression of anxiety (aOR, 2.76; 95% CI, 2.02-3.77) (Table 2).

**Table 1.** Rates of Postpartum Depression or Anxiety Following Stillbirth

Variable	No. (%)		*P* value	Odds ratio (95% CI)^b^	Adjusted odds ratio (95% CI)^c^
	Absence of paternal support (n = 31)^a^	Presence of paternal support (n = 238)			
Depression or anxiety	25 (80.6)	109 (45.8)	<.001	5.31 (3.18-8.87)	4.67 (2.73-8.00)
Depression alone	16 (51.6)	68 (28.6)	.02	2.60 (1.73-3.91)	2.23 (1.46-3.41)
Anxiety alone	18 (58.1)	74 (31.1)	.01	3.00 (1.99-4.54)	2.73 (1.75-4.26)

^a^ The pertinent yes or no question on the survey was, “Please let me know if you found the father of the baby helpful or supportive [time point].” This question was asked 3 times to reference 3 different time points: before the stillbirth, immediately after diagnosis of stillbirth, and 2 months following stillbirth.

^b^ Nonadjusted odds ratio from univariate logistic regression.

^c^ Adjusted for maternal race and ethnicity, prepregnancy depression and anxiety, and substance use during pregnancy in multivariate logistic regression. Prepregnancy depression and anxiety and substance use during pregnancy were self-reported during a predelivery interview.

**Table 2.** Risk Factors for Development of Maternal Postpartum Depression or Anxiety After Delivery of Stillborn Infant

Variable	Adjusted odds ratio (95% CI)^a^	*P* value
Lack of support from father of the stillborn infant	4.32 (2.46-7.60)	.009
Prepregnancy depression or anxiety	2.76 (2.02-3.77)	.001
Race (non-White)^b^	1.94 (1.37-2.76)	.07
Ethnicity (Hispanic)	1.86 (1.30-2.65)	.08
Chronic health conditions^c^	1.82 (1.32-2.51)	.06
Number of pregnancies	1.07 (0.96-1.18)	.52
Gestational age at stillbirth	1.00 (0.97-1.02)	.87
Maternal BMI	1.00 (0.98-1.03)	.90
Educational level, y	0.99 (0.91-1.06)	.84
Paternal age, y	0.99 (0.96-1.03)	.82
Maternal age, y	0.93 (0.89-0.98)	.14
Previous stillborn	0.90 (0.43-1.90)	.89
Previous pregnancy	0.80 (0.30-2.15)	.82

Abbreviation: BMI, body mass index.

^a^ Adjusted for all factors within table via multivariate analysis.

^b^ Including Asian or Pacific Islander, Black or African American, Native American or Alaskan Native, or other (defined in parent study as race not included as option in list of choices).

^c^ Defined as hypertension, asthma, seizure, diabetes, hyperthyroidism or hypothyroidism, heart disease, kidney disease, autoimmune disease, cancer, blood disorder, and chronic sexually transmitted infection.

## Discussion

An absence of perceived paternal support following stillbirth appears to be associated with markedly greater odds of maternal PPDA. Despite a diverse participant population and use of validated measures of PPDA as outcomes, this study has limitations: paternal support was defined dichotomously and there is risk of selection bias. Nevertheless, asking whether the father of a stillborn infant has been supportive may identify women at high risk for development of PPDA.
